# On the origin of fatty acid biosynthesis in *Archaeplastida*

**DOI:** 10.1093/jxb/erae462

**Published:** 2025-01-08

**Authors:** Amélie A Kelly, Ivo Feussner

**Affiliations:** Department for Plant Biochemistry, University of Goettingen, Albrecht-von-Haller-Institute for Plant Sciences, Justus-von-Liebig-Weg 11, D-37077 Goettingen, Germany; Department for Plant Biochemistry, University of Goettingen, Albrecht-von-Haller-Institute for Plant Sciences, Justus-von-Liebig-Weg 11, D-37077 Goettingen, Germany; Department for Plant Biochemistry, University of Goettingen, Goettingen Center for Molecular Biosciences (GZMB), Justus-von-Liebig-Weg 11, D-37077 Goettingen, Germany

**Keywords:** Endosymbiosis, fatty acid biosynthesis, glaucophytes, lipid transport

## Abstract

This article comments on:

**Sato N, Ikemura E, Uemura M, Awai K**. 2025. Genomic and biochemical analyses of lipid biosynthesis in *Cyanophora paradoxa*: limited role of the chloroplast in fatty acid synthesis. Journal of Experimental Botany **76**, 532–545. https://doi.org/10.1093/jxb/erae420

This article comments on:

**Sato N, Ikemura E, Uemura M, Awai K**. 2025. Genomic and biochemical analyses of lipid biosynthesis in *Cyanophora paradoxa*: limited role of the chloroplast in fatty acid synthesis. Journal of Experimental Botany **76**, 532–545. https://doi.org/10.1093/jxb/erae420

**A hallmark feature of photosynthetic organisms is the presence of fatty acid (FA) biosynthesis within plastids. This contrasts with mammals or fungi, where FAs are synthesized in the cytosol. Prompted by the lack of genes encoding a plastidial FA machinery, Sato *et al*. (2025) studied lipid biosynthesis in the alga *Cyanophora paradoxa* and found that isolated chloroplasts were indeed devoid of FA biosynthesis. This was corroborated by the identification of genes encoding cytosolic enzymes with homology to fungal Type I FATTY ACID SYNTHASE (FAS I). The results lead to open questions regarding the endosymbiotic origin and evolutionary advantages of establishing eukaryotic biosynthesis in this alga.**

The freshwater alga *Cyanophora paradoxa* belongs to the glaucophytes, which, together with the *Viridiplantae* (consisting of green algae and land plants) and the *Rhodophyta* (red algae) form a monophyletic clade known as *Archaeplastida* or *Plantae*. These constitute photosynthetic organisms with primary plastids; that is, eukaryotes that acquired a cyanobacterium in a single endosymbiosis event, as indicated by the presence of two envelope membranes, as opposed to other photosynthetic eukaryotes, in which ‘secondary’ plastids are mostly surrounded by four envelope membranes due to the acquisition of red or green algae. Glaucophyte plastids possess a peptidoglycan layer between the two envelope membranes, which is characteristic for cyanobacteria and other bacteria, but distinguishes the glaucophyte plastids from those found in the other lineages of the *Archaeplastida*. It has been shown to play a role in chloroplast division, and its presence is indicative of an early branching-off of the glaucophytes (Miyagishima *et al.*, 2014). With regards to the *Archaeplastidae*, primary endosymbiosis is used to describe a single event, by which a heterotrophic, unicellular eukaryote (also referred to as ‘host’ or ‘protist’) engulfed the ancestor of a cyanobacterium to gain photosynthetic capacity and ultimately evolve into a phototrophic organism. This was accompanied by endosymbiotic gene transfer of cyanobacterial genes to the host genome as well as permanent gene loss, which led to a drastic reduction of the donor genome. Gene loss also occurred on the recipient side, presumably to avoid gene redundancy. Lastly, the host genome harbors additional genes it acquired previously during the endosymbiotic incorporation of proteobacteria as predecessors of mitochondria and from other bacterial/unicellular genomes through horizontal gene transfer. Consequently, metabolic pathways, especially of primary metabolism, resemble a mix of prokaryotic and eukaryotic origin (Fernie *et al.*, 2024).

In plants, FA biosynthesis occurs in the plastid stroma (Fig. 1A). It is carried out in part by a FAS II which consists of several polypeptides (Harwood, 1996). In contrast, FAS I is a mono- or dimeric multidomain enzyme in the cytosol of fungi and animals (Leibundgut *et al.*, 2008). Respective proteins exhibit profound structural differences not only from those located in the chloroplast but also from each other. Plastid-derived FAs serve as building blocks for photosynthetic membranes, but they are also exported into the cytosol as precursors for eukaryotic glycerolipid assembly in the endoplasmic reticulum (ER). Selected lipid moieties are transported to other extraplastidial membranes and a substantial amount is even reimported into the plastids. As such, ‘photosynthetic’ lipid biosynthesis relies on extensive lipid trafficking across different compartments. Sato *et al.* (2025) compiled a comprehensive list of genes and enzymes implicated in lipid biosynthesis using comparative genomics based on the nuclear genome of *C. paradoxa* (Price *et al.*, 2019) and matched this with its lipidome. This worked well for all major lipid classes. Biosynthesis of phosphatidylcholine (PC), for example, appears to pass through methylation of phosphatidylethanolamine (PE) (as in red algae) and not through methylation of phosphoethanolamine (as in green algae).

**Fig. 1.**
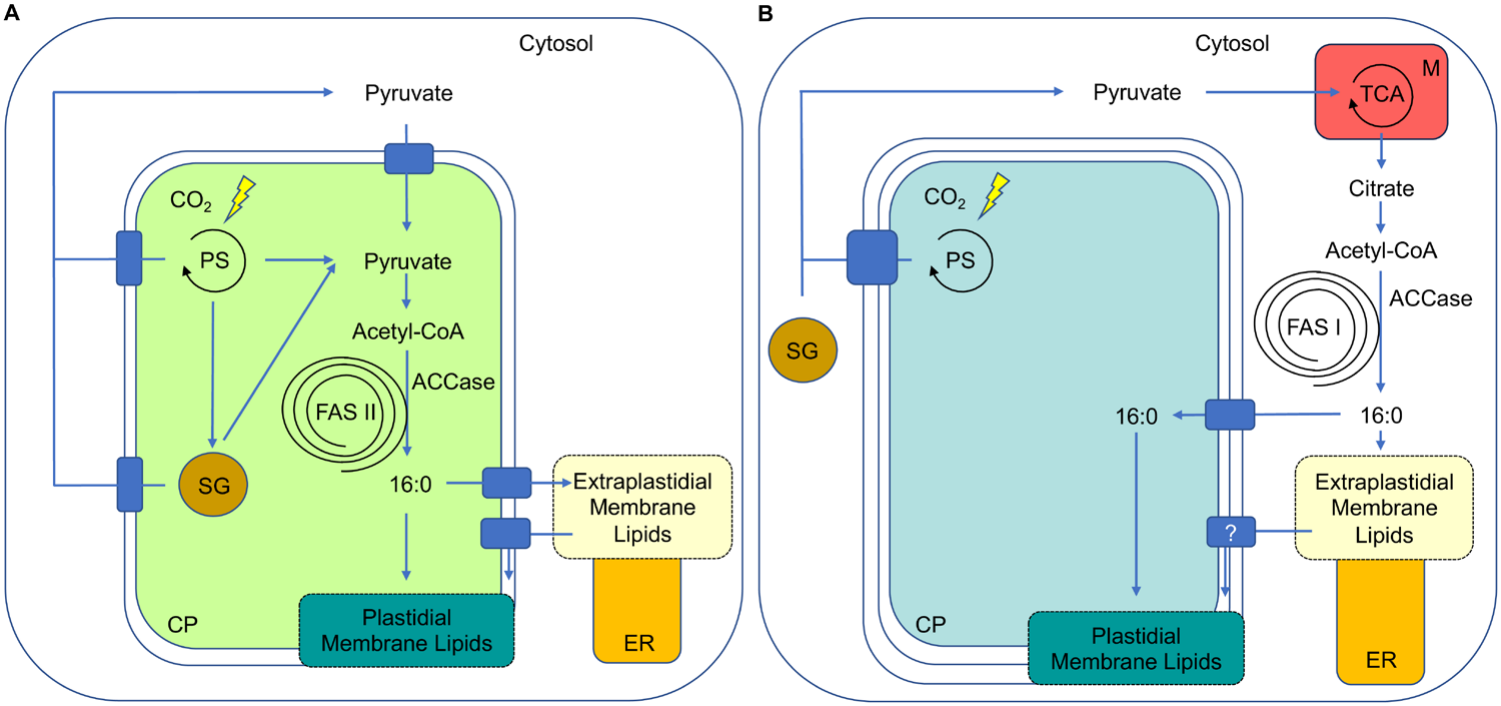
Simplified representation of the carbon flux from photosynthesis to membrane lipid biosynthesis in *Viridiplantae* and glaucophytes. (A) In *Viridiplantae de novo* synthesis of fatty acids (FAs) is fueled by carbon sources from photosynthesis and takes place inside the chloroplasts. Lipid moieties then either enter the plastidial route for membrane lipid biosynthesis, or they are exported to build extraplastidial lipids, some of which are reimported for synthesis of chloroplast lipids. (B) In glaucophytes, the entire FA biosynthesis takes place outside the chloroplast and relies on cytosolic carbon sources, which originate from photosynthesis. FAs are used for membrane lipid biosynthesis in the ER, or they are imported into chloroplasts for biosynthesis of plastidial membrane lipids. CP, chloroplast; M, mitochondrion; ER, endoplasmic reticulum; PS, photosynthesis; SG, starch granule; ACCase, acetyl-CoA carboxylase; FAS, fatty acid synthase; TCA, tricarboxylic acid.

The most intriguing observation though was the discovery of the cytosol as the only site of FA biosynthesis in *C. paradoxa* (Fig. 1B). This was inferred from the (i) apparent lack of genes encoding the expected prokaryotic acetyl-CoA carboxylase (ACCase) and key components of a plastidial FAS II in conjunction with the (ii) unexpected presence of genes encoding three cytosolic FAS I enzymes as well as the (iii) lack of any notable FA biosynthesis in isolated plastids during labeling experiments. The absence of FAS II was observed for two other glaucophytes (*Cyanoptyche gloeocystis* and *Gloeochaete witrockiana*) previously, indicating that this feature may be conserved among glaucophytes (Kohli *et al.*, 2016). In contrast, the other *Archaeplastida* not only integrated the *ACCase* and *FAS II* genes of the cyanobacterial progenitor into their host genome but redirected the whole FA machinery back to the plastids by equipping the respective proteins with target peptides. Transferring cyanobacterial genes into the nuclear genomes has been proposed to protect the genes from reactive oxygen species-induced damage or mutagenesis caused by physical proximity to photosynthesis (Kohli *et al.*, 2016). Further, establishing nuclear-encoded FA synthesis inside the chloroplast enables the host to coordinate FA biosynthesis and photosynthesis through transcriptional control of gene expression as well as *in situ* as observed for plant ACCase (Ohlrogge and Jaworski, 1997).

The ancestors of the *Archaeplastida* harbor isoprenoid-based ether lipids and not FA-containing lipids in their membranes, which implies that at some point not only *FAS II*, but also *FAS I* genes must have been acquired, though the exact order and partners are unclear (Matsumi *et al.*, 2011). Also, a functional advantage for *C. paradoxa* to lose the cyanobacterial genes but retain the eukaryotic FAS machinery remains to be demonstrated. One may speculate that the subcellular supply or level(s) of energy and carbon for FA synthesis played a role: in plants, plastidial FA synthesis is fueled by ATP and NADPH from photosynthesis, whereas the carbon substrates to generate the required acetyl-CoA moieties are provided by pyruvate from cytosolic glycolysis. For this, photosynthetically fixed carbon is incorporated during the light period into triose phosphates and exported for sucrose synthesis in the cytoplasm, or it is used for starch biosynthesis inside the chloroplast, which in turn is degraded and exported to sustain plant growth and maintenance during the night. In *C. paradoxa*, ATP citrate lyase is a cytosolic enzyme and generates acetyl-CoA from mitochondrial-derived citrate (Ma *et al.*, 2001). In contrast to green algae and land plants, which synthesize starch from ADP-glucose, glaucophytes and red algae synthesize starch through a UDP-glucose pathway and store it as starch granules in the cytosol, resulting in a substantially altered carbon flux in these organisms (Plancke *et al.*, 2008).

The analyses performed with isotopes by Sato *et al.* (2025) allow a clear differentiation between *de novo* FA synthesis and further processing of existing FA moieties. In conjunction with their comparative genomics approach, the authors constructed a comprehensive model of a mostly cytosolic desaturation and elongation pathway for the newly synthesized FAs. In line with this, the overall FA composition of both chloroplast and extrachloroplast lipids was similar, supporting a common origin. The FAs 16:0 (30–70%) and, to a lesser extent, 20:4 and 20:5 (10–30 %) accounted for almost all FAs in the glycerolipids. This is in stark contrast to glycerolipids of land plants, for example, where the FA composition is determined by distinct substrate specificities of the respective acyltransferases (Bates *et al.*, 2013). It is not only highly indicative of the biosynthetic pathways per se (plastid versus cytosol/ER or across both compartments), but it also reflects membrane lipid remodeling and transport between different lipid pools in the cell during adverse growth conditions. Despite similar FA profiles for plastidial and extraplastidial lipids in *C. paradoxa*, positional analyses revealed differences in the distribution of FAs on the *sn*-1 or *sn*-2 position of glycerol. Together with the identification of sequences for the respective candidate acyltransferases in both compartments, this may suggest that not glycerolipids, but single FA moieties are imported into the chloroplast before being incorporated into plastidial glycerolipid biosynthesis. In Arabidopsis, substantial lipid import into chloroplasts has been described mostly for complex lipids such as PC, phosphatidic acid (PA), or diacylglycerol (DAG) as precursors of galactolipid biosynthesis. The TRIGALACTOSYLDIACYLGLYCEROL complex and more recently Sec13 proteins have been reported to mediate primarily PA import into the chloroplast, whereas FAX1 (FATTY ACID EXPORT 1) has been suggested to be involved in exporting FAs from the chloroplast to the cytosol (Li *et al.*, 2016). LACS9, a member of the long-chain acyl-CoA synthetase family (LACS), which convert FAs to their corresponding CoA thioesters to become metabolically available, is involved in FA import (18:2) into the plastid (Jessen *et al.*, 2015). All represent valid candidates for importing FAs into the chloroplast in glaucophytes.

Lastly, the observation that glaucophytes appear to have retained their protist-derived (eukaryotic) FAS I is reminiscent of galactolipid biosynthesis. Cyanobacteria are rich in galactolipids, which represent the major components of photosynthetic membranes, a feature cyanobacteria share with other photosynthetic organisms, and which distinguishes them from most other non-photosynthetic organisms. Consequently, the presence of galactolipids in plants is often described as of being cyanobacterial origin. Interestingly, however, a large number (but not all) of the biosynthesis genes within the *Archaeplastida* are of eukaryotic origin and lack sequence similarity-based homologs to those of free-living cyanobacteria (Sato and Awai, 2016). This suggests that they must have been present and functional before the *Archaeplastida* split into the three different groups, possibly even before the actual primary endosymbiosis occurred (Sato, 2020, 2021). Based on these investigations regarding the extent and the chronology of endosymbiotic events, Sato *et al*. (2025) put forward a hypothesis, where the protist already synthesized galactolipids to cope with phosphate-limiting growth conditions, and galactolipid-rich membranes may have played a key role in acquiring the donor.

In summary, lipid biosynthesis in *C. paradoxa* displays distinct features with regards to its phylogenetic origin and subcellular location. Further investigations into the specific physiological needs or possibly the life cycle of glaucophytes may elucidate the evolutionary advantage for the spatial rearrangements of lipid and starch metabolism as well as underlying evolutionary advantages favoring eukaryotic over cyanobacterial pathways despite similar functions.
